# Cell-Specific Cre Recombinase Expression Allows Selective Ablation of Glutamate Receptors from Mouse Horizontal Cells

**DOI:** 10.1371/journal.pone.0083076

**Published:** 2013-12-12

**Authors:** Sebastian Ströh, Stephan Sonntag, Ulrike Janssen-Bienhold, Konrad Schultz, Kerstin Cimiotti, Reto Weiler, Klaus Willecke, Karin Dedek

**Affiliations:** 1 Department of Neurobiology, University of Oldenburg, Oldenburg, Germany; 2 Research Center Neurosensory Science, University of Oldenburg, Oldenburg, Germany; 3 Life and Medical Sciences Institute, University of Bonn, Bonn, Germany; Lancaster University, United Kingdom

## Abstract

In the mouse retina, horizontal cells form an electrically coupled network and provide feedback signals to photoreceptors and feedforward signals to bipolar cells. Thereby, horizontal cells contribute to gain control at the first visual synapse and to the antagonistic organization of bipolar and ganglion cell receptive fields. However, the nature of horizontal cell output remains a matter of debate, just as the exact contribution of horizontal cells to center-surround antagonism. To facilitate studying horizontal cell function, we developed a knockin mouse line which allows ablating genes exclusively in horizontal cells. This knockin line expresses a Cre recombinase under the promoter of connexin57 (Cx57), a gap junction protein only expressed in horizontal cells. Consistently, in Cx57+/Cre mice, Cre recombinase is expressed in almost all horizontal cells (>99%) and no other retinal neurons. To test Cre activity, we crossbred Cx57+/Cre mice with a mouse line in which exon 11 of the coding sequence for the ionotropic glutamate receptor subunit GluA4 was flanked by two *loxP* sites (GluA4fl/fl). In GluA4fl/fl:Cx57+/Cre mice, GluA4 immunoreactivity was significantly reduced (∼50%) in the outer retina where horizontal cells receive photoreceptor inputs, confirming the functionality of the Cre/*loxP* system. Whole-cell patch-clamp recordings from isolated horizontal cell somata showed a reduction of glutamate-induced inward currents by ∼75%, suggesting that the GluA4 subunit plays a major role in mediating photoreceptor inputs. The persistent current in GluA4-deficient cells is mostly driven by AMPA and to a very small extent by kainate receptors as revealed by application of the AMPA receptor antagonist GYKI52466 and concanavalin A, a potentiator of kainate receptor-mediated currents. In summary, the Cx57+/Cre mouse line provides a versatile tool for studying horizontal cell function. GluA4fl/fl:Cx57+/Cre mice, in which horizontal cells receive less excitatory input, can thus be used to analyze the contribution of horizontal cells to retinal processing.

## Introduction

Horizontal cells are interneurons in the mammalian retina which receive glutamatergic input from photoreceptors via ionotropic glutamate receptors [1]. In turn, horizontal cells provide feedback and feedforward signals to photoreceptors and bipolar cells, respectively [2], allowing the retina to adjust to a broad range of light intensities. The mouse retina only contains a single type of horizontal cell - the axon-bearing B-type [3], which forms axo-axonal and dendro-dendritic networks coupled by the gap junction-forming protein connexin57 (Cx57) [4]–[6]. Although it is well known that horizontal cells play an important role in formation and maintenance of triad synapses with photoreceptors and bipolar cells [7] and in gain control of this synapse [8], many aspects of horizontal cell function remain elusive, e.g. the nature of the negative and positive feedback signals to rods and cones or the contribution of horizontal cells to ganglion cell receptive fields. Different techniques have been used to study horizontal cell function, including pharmacological approaches [9]–[12], knockout mouse models [5], [13], [14], horizontal cell ablation by kainate [15]–[17], or the diphtheria toxin (DT)/DT receptor system [7]. However, pharmacological approaches are often difficult because blockers of ion channels tend to affect many retinal circuits as is also often the case with knocking out retinal proteins. Selective killing of horizontal cells led to severe remodeling and disruption of the first visual synapse [7], [15]–[17] and is only partly suitable to study the functional role of horizontal cells in the mouse retina.

Here, we introduce a new mouse model as a tool for studying horizontal cell function. It allows the selective deletion of individual proteins from horizontal cells using a horizontal cell-specific Cre recombinase. The Cre/*loxP* system is based on a P1 bacteriophage protein, binding to a target recognition site that is 34 bp long and known as *loxP*. Cre recombinase is able to excise *loxP*-flanked (floxed) sequences from the genome [18], [19]. To drive Cre expression selectively in horizontal cells, a cell-specific promoter was needed that is not active elsewhere in the retina and (ideally) no other tissue. The Cx57 promoter, which regulates expression is of the major protein subunit of gap junction channels between mouse horizontal cells [6], fulfills this requirement. It is only active in retinal horizontal cells, the thymus medulla, and embryonic kidney [4], [7], [20] and potentially in the cerebellum [21]. Thus, we used the promoter of Cx57 [20] to drive Cre expression. To test the suitability of the resulting Cx57-Cre mouse line as a tool for studying horizontal cells, we crossbred it with a mouse line in which exon 11 of the coding sequence for the ionotropic glutamate receptor subunit GluA4 was flanked by two *loxP* sites. This led to the deletion of the first two transmembrane regions of the GluA4 subunit [22] exclusively in horizontal cells. Success and specificity of GluA4 ablation were demonstrated using quantitative immunohistochemistry, Western blot, and patch-clamp recordings from isolated horizontal cells.

## Results

### Generation of a horizontal cell-specific Cre-expressing mouse line

**Figure 1:**
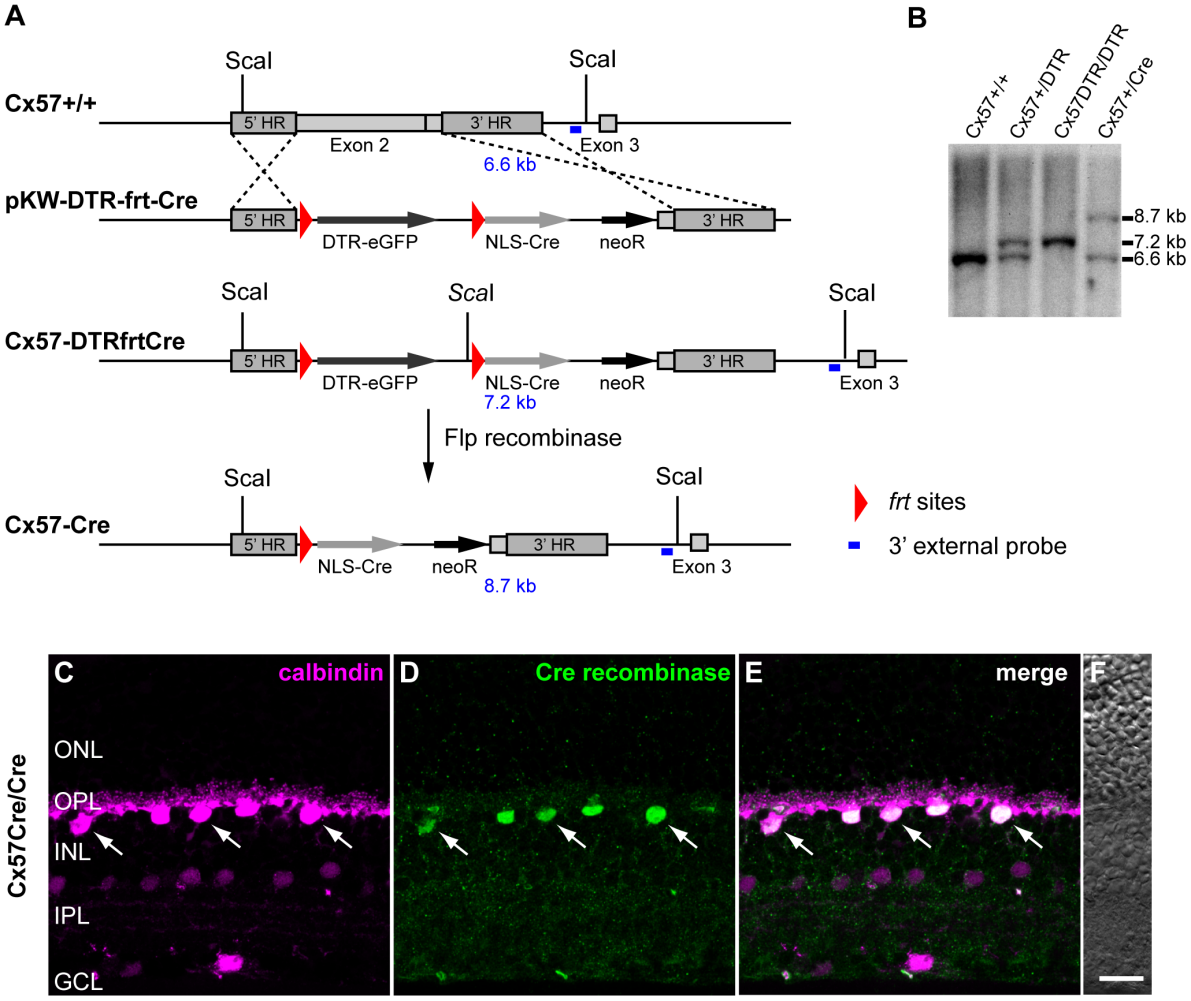
Generation of Cx57-Cre mice and exclusive Cre expression in horizontal cells. ***A***, Targeting scheme. The coding region of the wild-type *Cx57* gene is located in exon 2 and 3. After electroporating the targeting vector (pKW-DTR-fret-Cre), a large part of exon 2 was replaced by a DTR-eGFP construct, flanked by *frt* sites, and the coding sequence for the NLS-Cre recombinase followed by a neomycin resistance cassette (*neoR*). After Flp- mediated recombination, the coding sequence for the NLS-Cre recombinase gets under the control of the Cx57 promoter. DTR-eGFP: fusion protein of the diphtheria toxin receptor (DTR) and eGFP; NLS: nuclear localization signal. ***B***, Southern blot analysis of ScaI-digested genomic DNA to test for correct recombination. A signal of 7.2 kb confirms the correct targeting of the Cx57-DTRfrtCre construct to the *Cx57* locus. The functioning of the *frt* sites and successful Flp-mediated excision of DTR were verified by a signal of 8.7 kb. ***C-E***, Projections of confocal stacks of vertical retina sections from Cx57Cre/Cre mice labeled with antibodies against calbindin (magenta; C, E), a marker for mouse horizontal cells, and Cre recombinase (green; D, E). Only calbindin-positive horizontal cells show Cre recombinase immunoreactivity in their nuclei (arrows) in the distal INL; no other retinal cell type is Cre-positive. ***F***, The Nomarski image shows the normal retinal layering. ONL/INL, outer/inner nuclear layer; OPL/IPL, outer/inner plexiform layer; GCL, ganglion cell layer. Scale bar: C-F, 20 µm.

To generate a horizontal cell-specific Cre recombinase-expressing mouse line, we used the Cx57 promoter to drive Cre expression [4]. As described previously [7], we generated a targeting vector pKW-DTR-frt-Cre and replaced part of the *Cx57* coding sequence in exon 2 with the *frt*-flanked coding sequence of *DTR-eGFP* followed by the coding sequence of Cre recombinase (Fig. 1A). This approach permitted the *Cx57* promoter-dependent expression of the DTR and thus selective ablation of horizontal cells via DT injection [7]. Moreover, it also allowed Flp-mediated excision of *DTR-eGFP*, leaving the Cre recombinase under the control of the *Cx57* promoter. For selection of embryonic stem cells, a neomycin resistance gene under the control of the phosphoglycerate kinase promoter (PGK-neo) was used (Fig. 1A). To confirm correct Flp-mediated recombination in the *Cx57* locus, Southern blot analysis was performed using ScaI-digested genomic DNA from liver tissue. Hybridization with a 500 bp probe (Fig. 1A, blue line) corresponding to a region in intron 2 of *Cx57* downstream of the 3’ homology region resulted in the expected signals (Cx57 wild-type allele: 6.6 kb; Cx57Cre allele: 8.7 kb) and indicated correct recombination (Fig. 1B).

### Retinal Cre recombinase expression is selective for horizontal cells

To control for expression in horizontal cells of the mouse retina, we double labeled vertical sections from mice expressing two alleles of Cx57-Cre (Cx57Cre/Cre mice) with antibodies against Cre recombinase and calbindin (Fig. 1C, D), a marker for horizontal, amacrine and ganglion cells [23]. Cre expression was restricted to horizontal cells, as revealed by the colocalization with calbindin in the distal inner nuclear layer (INL) where horizontal cell bodies reside (Fig. 1E, arrows). Retinal layering was normal (Fig. 1F) and no other cell type was labeled in Cx57Cre/Cre retinas (Fig. 1E), confirming the cell specificity of Cre expression.

### Cre expression can be used to ablate the glutamate receptor subunit GluA4 from horizontal cells

**Figure 2:**
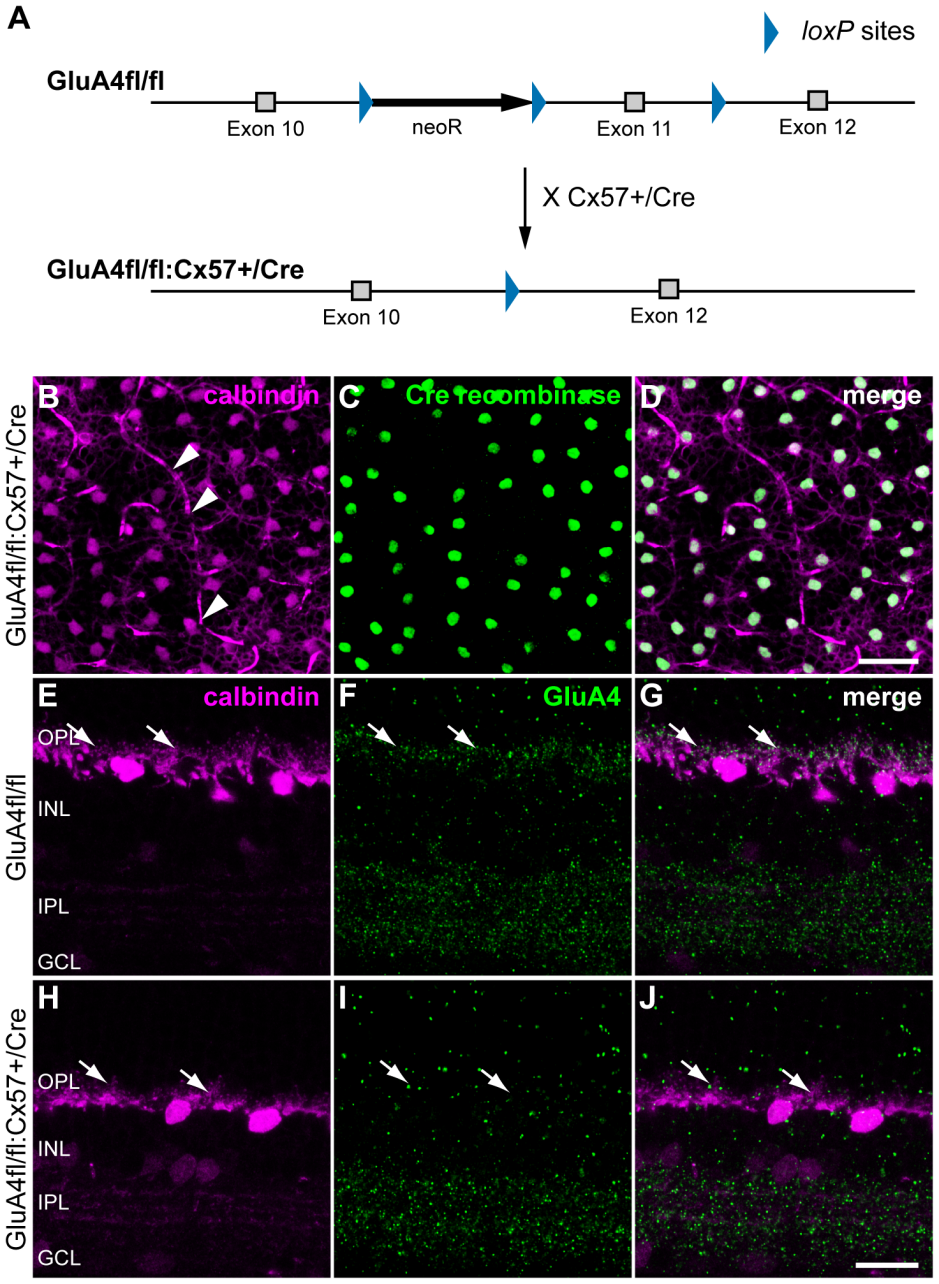
Cre expression in GluA4fl/fl:Cx57+/Cre mice leads to reduced GluA4 immunoreactivity in the outer retina. ***A***, Generation of the conditional GluA4-deficient mice by crossing Cx57+/Cre mice with GluA4fl/fl mice [22]. Expression of Cre recombinase leads to Cre-mediated ablation of the GluA4 gene only in Cx57-positive cells. ***B-D***, Confocal projections of the OPL and distal INL from a GluA4fl/fl:Cx57+/Cre mouse labeled for calbindin (magenta; B, D) and Cre recombinase (green; C, D). Each calbindin-positive horizontal cell is also positive for Cre recombinase. Arrowheads point to blood vessels labeled by unspecific binding of the secondary anti-mouse antibody. ***E-J***, Double labeling for calbindin (magenta) and GluA4 (green) in GluA4fl/fl (E-G) and GluA4fl/fl:Cx57+/Cre mice (H-J) confirmed the selective deletion of GluA4 in the OPL (arrows) but not the IPL of GluA4fl/fl:Cx57+/Cre mice. OPL/IPL, outer/inner plexiform layer; INL, inner nuclear layer; GCL, ganglion cell layer. Scale bars: B-D, 40 µm; E-J: 20 µm.

Several studies have shown that rodent horizontal cells express different types of ionotropic glutamate receptors [1], [9], including α-amino-3-hydroxy-5-methyl-4-isoxazolepropionic acid (AMPA) receptor subunits GluA1-4 [1], and kainate receptors (GluK1-3) [24], but not N-methyl-D-aspartate (NMDA) receptors [9]. However, the contribution of an individual receptor subunit to the glutamate response of horizontal cells is not known. Thus, to test whether the Cre recombinase enzyme is functional in horizontal cells and the system suitable for horizontal cell characterization, we crossbred Cx57+/Cre mice with floxed GluA4 mice (kind gift of Dr. Elke Fuchs and Prof. Hannah Monyer, University of Heidelberg, Heidelberg, Germany) [22] to selectively ablate this AMPA receptor subunit from horizontal cells (Fig. 2A). Heterozygous Cx57+/Cre mice were chosen to minimize side effects from Cx57 deficiency [5].

First, we tested whether Cre expression in horizontal cells is patchy or uniform across the retina of GluA4fl/fl:Cx57+/Cre mice. To this end, we quantified the number of calbindin-positive horizontal cells that were also Cre-positive. As illustrated in Figure 2B-D, we found an almost perfect overlap of Cre recombinase expression and calbindin labeling in retinal whole-mounts. Quantifications from 16 retinal fields from two different GluA4fl/fl:Cx57+/Cre mice showed that 99.98±0.5% of the calbindin-positive horizontal cells are also positive for Cre recombinase. Similar results were obtained for Cx57Cre/Cre mice (not shown). Horizontal cell densities (GluA4fl/fl:Cx57+/Cre: 1,024±233 cells/mm^2^; Cx57+/Cre: 1,234±177 cells/mm^2^) were comparable to other mouse lines [25]. Thus, unlike in many other Cre-expressing transgenics [19], [26], expression of Cre recombinase is uniform and not mosaic in GluA4fl/fl:Cx57+/Cre mice. This may be due to the insertion into a previously characterized locus [4].

Next, we examined whether the recombinase is enzymatically effective and able to efficiently excise GluA4 from horizontal cell genomic DNA. Double immunostaining for calbindin (Fig. 2E, G) and GluA4 (Fig. 2F, G) revealed strong GluA4 immunoreactivity in the outer plexiform layer (OPL) of control mice (GluA4fl/fl) where horizontal cell dendrites and axon terminals invaginate photoreceptor endings and receive glutamatergic inputs. In GluA4fl/fl:Cx57+/Cre mice, in contrast, GluA4 immunoreactivity was strongly reduced in the OPL (Fig. 2H-J, arrows) but was comparable to controls in the inner plexiform layer (IPL). This shows that the Cx57-driven Cre enzyme eliminated GluA4 from horizontal cells but not from other retinal neurons, again demonstrating the specificity of the system.

**Figure 3:**
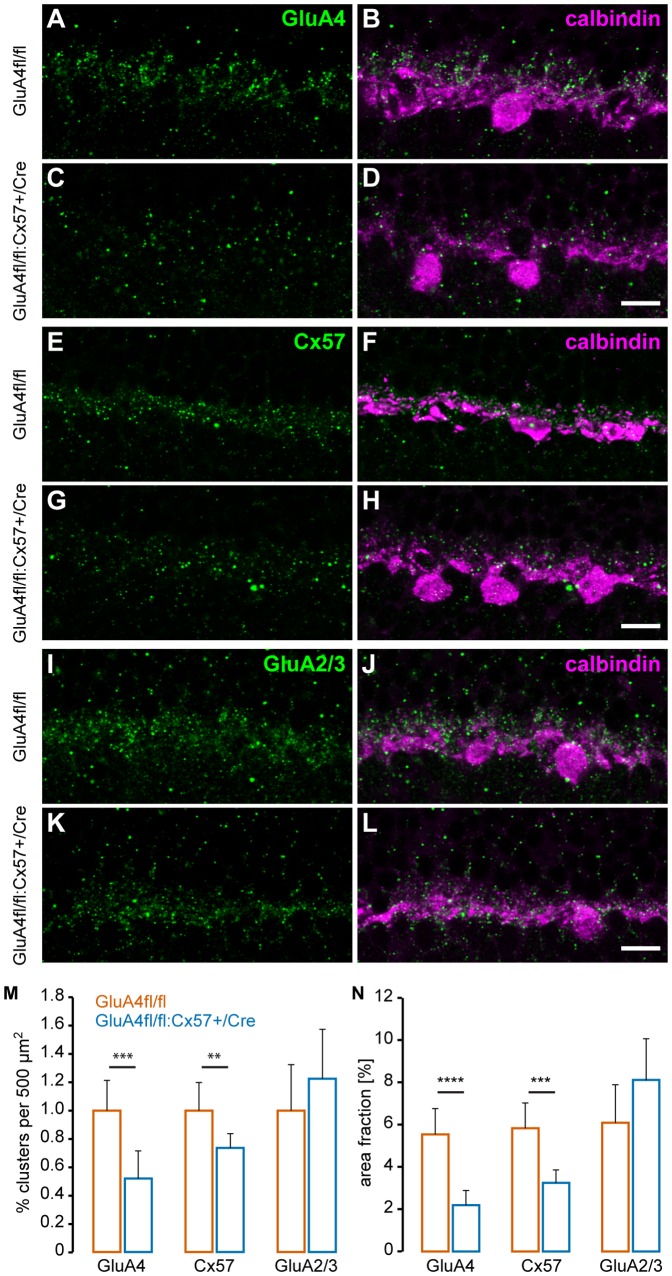
GluA4 and Cx57 expression are reduced in GluA4fl/fl:Cx57+/Cre mice. ***A-L***, Expression of GluA4 (A-D), Cx57 (E-H), and GluA2/3 (I-L) were quantified in mice in GluA4-expressing (A, B, E, F, I, J) and GluA4-deficient horizontal cells (C, D, G, H, K, L) labeled with anti-calbindin antibodies (B, D, F, H, J, L). Maximum projections of two confocal images are shown. ***M***, The number of immunoreactive puncta per 500 µm^2^ was determined in both genotypes and normalized to the counts in GluA4fl/fl control mice. Cx57 and GluA4 immunoreactivity were reduced in GluA4-deficient horizontal cells (GluA4fl/fl:Cx57+/Cre) while GluA2/3 immunoreactivity was not significantly different between genotypes. Similar results were obtained when the area fraction was determined that was occupied by immunoreactive puncta (N). Values in M and N are given as mean ± SD. **, p < 0.01; ***, p < 0.001; ****, p < 0.0001. n = 6 to 10. Scale bars: A-L: 20 µm.

To further quantify this, we determined the number of GluA4-immunoreactive puncta in control and Cre-expressing retinas (Fig. 3A-D, M). Data averaged from at least seven regions of interest (ROI) per genotype (Fig. 3M) revealed a highly significant reduction (48±19%) in the number of GluA4 clusters in the OPL of GluA4fl/fl:Cx57+/Cre mice (p = 2.9×10^−4^). To exclude that clusters were reduced in numbers but increased in size, we also calculated the area fraction that GluA4 clusters occupied in the specified ROI. This area fraction was also strongly and significantly reduced (60±12%) in Cre-expressing mice (p = 3.5×10^−6^), indicating that GluA4 immunoreactivity in the OPL is strongly reduced (Fig. 3N).

As Cre-expressing mice possess only one intact allele of *Cx57*, we expected the immunoreactivity for Cx57 to decrease as well. This was indeed the case: the number of clusters and the area fraction were significantly reduced when compared to control mice (Fig. 3E-H, M, N; number of clusters: p = 0.0082; area fraction: p = 2.7×10^−4^).

We also quantified the GluA2/3 immunoreactivity in the OPL of control and Cre-expressing mice (Fig. 3I-L, M, N) to test for an up-regulation of other AMPA receptor subunits known to be expressed in rodent horizontal cells [1]. Although GluA2/3 immunoreactivity showed the tendency to be more prominent in the OPL of GluA4fl/fl:Cx57+/Cre mice, differences between genotypes were not significant (number of clusters: p = 0.246; area fraction: p = 0.073).

**Figure 4:**
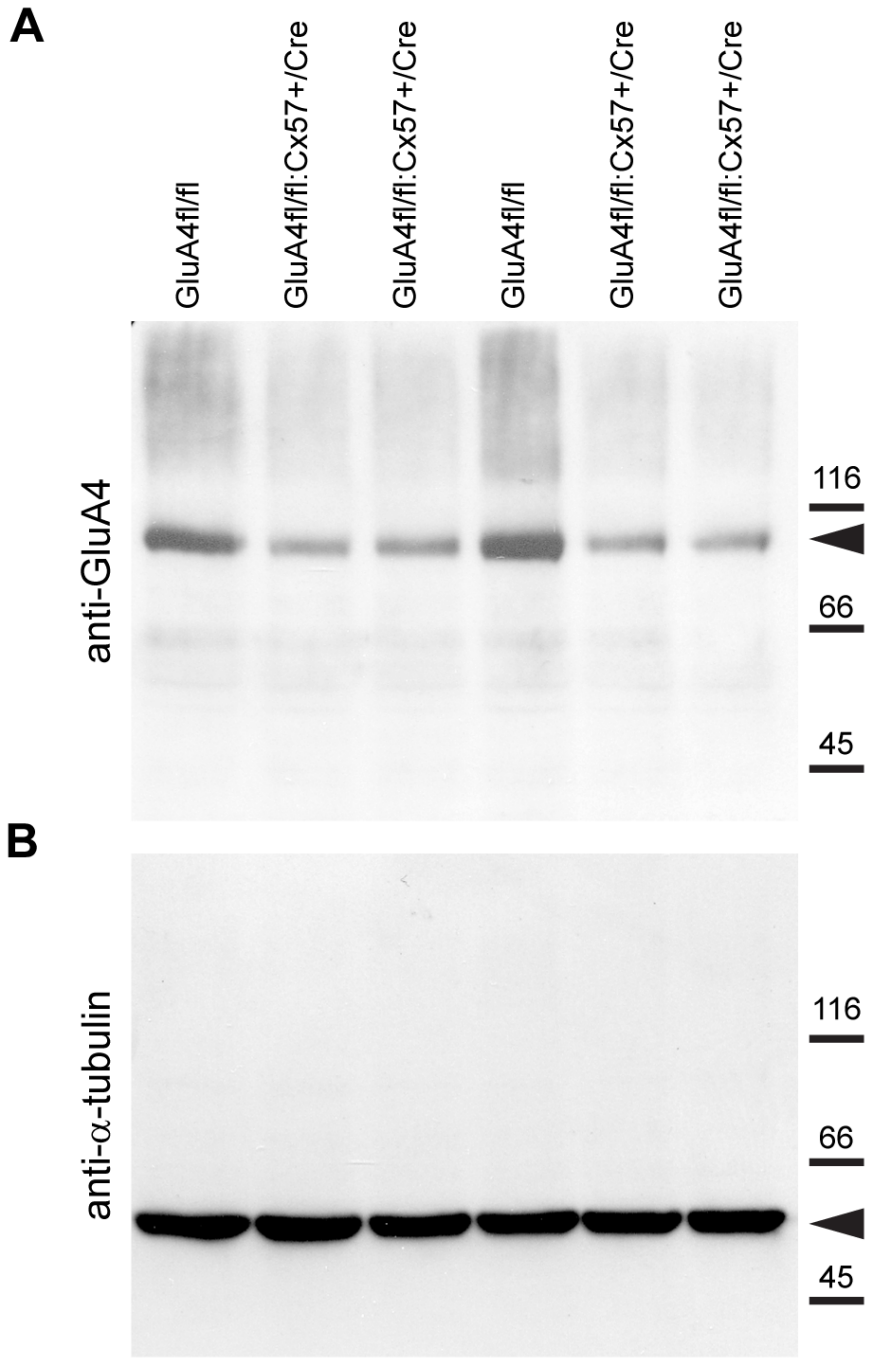
GluA4 protein levels are reduced in retina homogenates from GluA4fl/fl:Cx57+/Cre mice. ***A***, Western blot analysis of GluA4 expression in retina homogenates from GluA4fl/fl and GluA4fl/fl:Cx57+/Cre mice. GluA4-specific antibodies detected a single protein band migrating at ∼100 kDa (arrowhead). Expression levels are lower in GluA4-deficient mice than in controls. ***B***, To control for total protein amount, the blot was stripped and incubated with antibodies against the housekeeping protein α-tubulin. A single immunoreactive band of ∼50 kDa was detected (arrowhead) revealing that protein amounts were comparable in homogenates from both genotypes. Molecular mass marker proteins are indicated on the right (kDa).

Ablation of GluA4 in GluA4fl/fl:Cx57+/Cre retinas was also confirmed by Western blot analysis of total retina homogenates from GluA4fl/fl and GluA4fl/fl:Cx57+/Cre mice. Western blots derived from 8–10% gradient SDS-PAGE gels, on which equal amounts (50 µg) of retinal proteins of GluA4fl/fl and GluA4fl/fl:Cx57+/Cre retinas were separated, showed a prominent reduction in GluA4-immunoreactivity (∼100 kDa) in all GluA4fl/fl:Cx57+/Cre samples (Fig. 4A, arrowhead). In contrast, immunoreactivity of the reference protein α-tubulin was not affected (Fig. 4B, arrowhead). The density of the GluA4-immunoreactive protein (Fig. 4A) was quantified in relation to the α-tubulin-immunoreactive protein (∼50 kDa) which served as a loading control. In GluA4fl/fl:Cx57+/Cre mice (n = 7 mice), the density of GluA4 bands was significantly reduced by 47±15% (p = 0.0026) when compared to controls (n = 5 GluA4fl/fl mice) and consistent with the selective deletion of GluA4 from horizontal cells, but not from neurons in the IPL (Fig. 2H-J).

### Horizontal cells from GluA4fl/fl:Cx57+/Cre mice show reduced glutamate-induced currents

**Figure 5:**
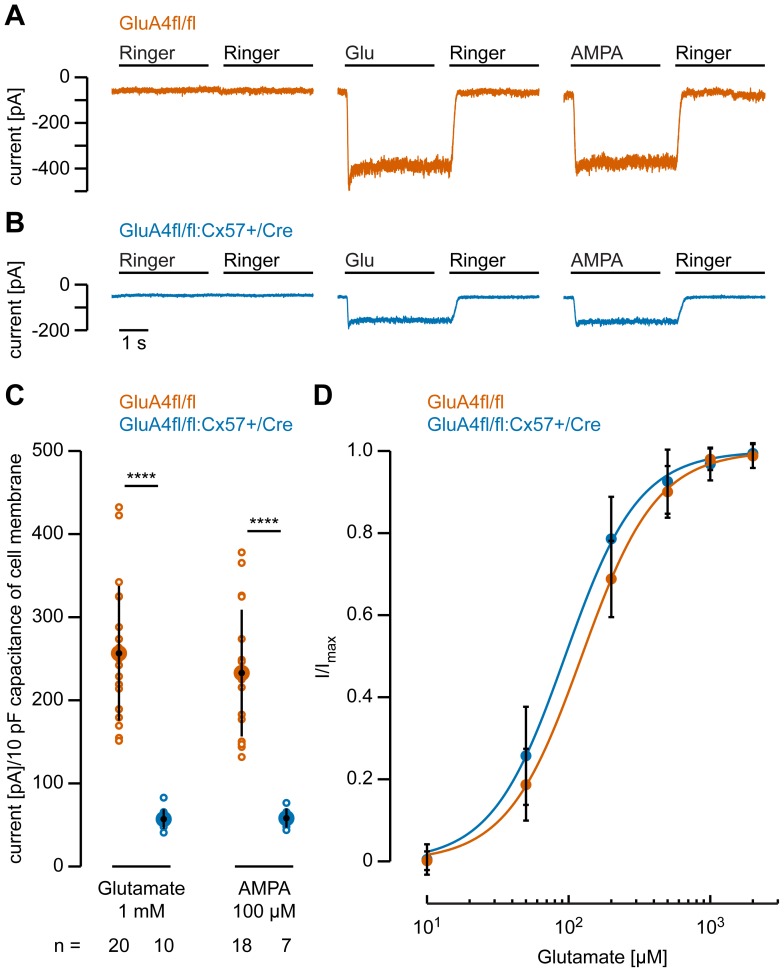
Glutamate- and AMPA-induced currents are reduced in horizontal cells lacking the GluA4 subunit. ***A***, ***B***, Representative current traces recorded from GluA4-expressing (A) and GluA4-deficient horizontal cell somata (B) at a holding potential of -70 mV. No significant inward current was elicited by Ringer’s solution. However, inward currents were induced by glutamate (Glu, 1 mM) or AMPA application (100 µM). Drug-induced currents were still present but strongly reduced in GluA4-deficient horizontal cells (B). ***C***, Quantification of the glutamate- and AMPA-induced currents in both genotypes. In GluA4-deficient horizontal cells, current amplitudes were significantly reduced by about 75% compared to control cells. Please note that current amplitudes are normalized to 10 pF capacitance of cell membrane to account for differences in cell size. Small circles represent values for individual cells whereas large, black-filled circles represent the means; error bars show SD. n specifies the number of measured cells. ***D***, Dose-response curve for glutamate, measured for both genotypes (GluA4fl/fl: n = 11; GluA4fl/fl:Cx57+/Cre: n = 7). Data were normalized to I_max_ and fitted to the Hill equation to obtain EC_50_ values (GluA4fl/fl: 128 ±38 µM; GluA4fl/fl:Cx57+/Cre: 99 ±32 µM) and Hill coefficients (GluA4fl/fl: 1.71 ±0.15; GluA4fl/fl:Cx57+/Cre: 1.81 ±0.57). Differences were not significant (EC_50_: p = 0.12; Hill: p = 0.56). Values are given as mean ± SD. ****, p < 0.0001.

To test whether the deletion of the GluA4 subunit affected the glutamate response of horizontal cells, we performed whole-cell patch-clamp recordings from isolated horizontal cell somata of both genotypes [5], [9]. Isolated horizontal cells were visually identified according to their characteristic morphology [9]: a polygonal soma with a size of ∼11 µm and two to five primary dendrites. Currents were measured at a holding potential of –70 mV. As we used a slow drug application system, only desensitized currents were measured [27]. To ensure maximal current amplitudes, long drug application times (≥3 s) were chosen. Ringer’s solution was applied to accelerate the washout of agonists or antagonists. To account for differences in cell size and membrane surface, currents were normalized to the membrane capacitance. In GluA4fl/fl control mice, glutamate induced an inward current (Fig. 5A) with a mean amplitude of about 257±81 pA/10 pF membrane capacitance (Fig. 5C). Application of 100 µM AMPA gave rise to an inward current with similar amplitude (233 ±76 pA/10 pF membrane capacitance; Fig. 5A, C). In contrast, in GluA4-deficient mice, glutamate elicited significantly smaller inward currents with a mean amplitude of 57 ±12 pA/10 pF membrane capacitance (Fig. 5B, C; p = 2.32×10^−8^). A similar reduction of ∼75% was found when AMPA was applied (p = 4.51×10^−6^). Thus, GluA4 contributes considerably to the glutamate-induced conductance in horizontal cell somata. However, as ∼25% of the current persisted, glutamate receptors must still be present in horizontal cells from GluA4fl/fl:Cx57+/Cre mice. To test whether the glutamate affinity of these receptors differed from receptors in control cells, we determined a glutamate dose-response curve for both genotypes (Fig. 5D) and compared the EC_50_ values and Hill coefficients. However, we found no differences between genotypes: EC_50_ values were 128 ±38 µM for GluA4fl/fl mice and 99 ±32 µM for GluA4fl/fl:Cx57+/Cre mice (p = 0.12), respectively. Hill coefficients of 1.71 ±0.15 (control) and 1.81 ±0.57 (GluA4-deficient) were also similar (p = 0.56) and fitted well with previous reports [26].

**Figure 6:**
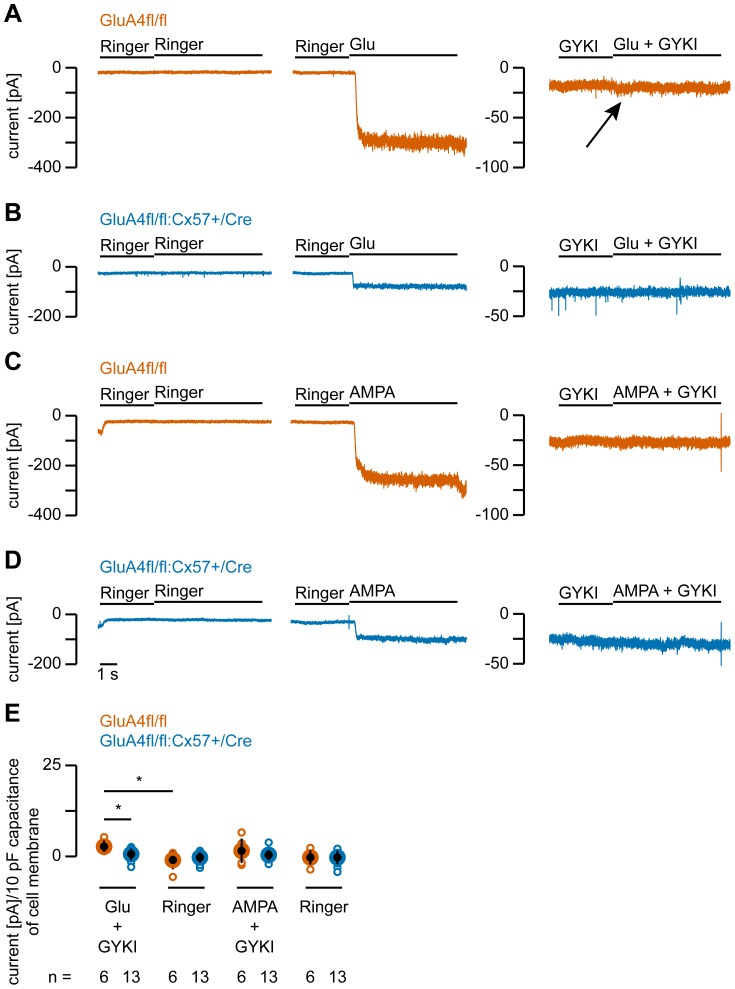
The residual glutamate-induced current in GluA4-deficient horizontal cells is mainly mediated by AMPA receptors. ***A-D***, Representative current traces recorded from GluA4-expressing (A, C) and GluA4-deficient horizontal cell somata (B, D) at a holding potential of –70 mV. When the AMPA receptor antagonist GYKI52466 (100 µM) was present, glutamate (Glu, 1 mM; A, B, E) only induced a detectable current in GluA4-expressing cells (A,E) but not in GluA4-deficient cells (B, E). AMPA-induced responses were completely abolished by GYKI52466 in both genotypes (C-E). ***E***, Quantification of glutamate- and AMPA-induced currents upon GYKI52466 application. GYKI52466 reduced the amplitudes of glutamate- and AMPA-induced currents by 98.9% in controls and 99.3% in GluA4-deficient mice, indicating that most of the glutamate-induced current is mediated by AMPA receptors. Current amplitudes in E are normalized to 10 pF capacitance of cell membrane to account for differences in cell size. Small circles represent values for individual cells; large, black filled circles represent the means; error bars show SD. n specifies the number of measured cells. *, p < 0.05.

To determine the type of glutamate receptor that mediates the residual glutamate-induced current in GluA4fl/fl:Cx57+/Cre mice, we applied the AMPA receptor blocker GYKI52466 (100 µM) in the presence of glutamate (Fig. 6A,B,E) or AMPA (Fig. 6C-E). In GluA4fl/fl controls (Fig. 6A,C), GYKI52466 almost completely blocked the currents induced by glutamate (98.9%) and AMPA (99.3%). However, for glutamate a very small current persisted (Fig. 6A, arrow) which was significantly different from control responses to Ringer’s application (Fig. 6E; glutamate: p = 0.0101). In GluA4fl/fl:Cx57+/Cre cells, glutamate- and AMPA-induced inward currents were completely abolished by GYKI52466 (Fig. 6B,D,E).

**Figure 7:**
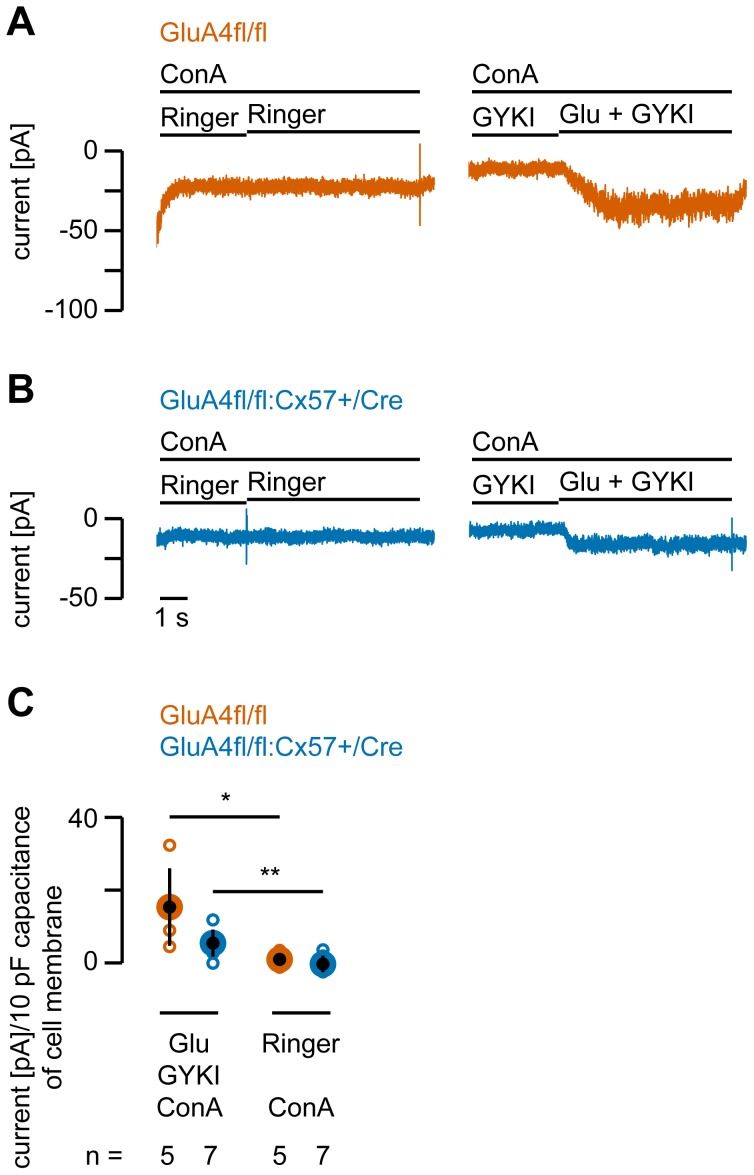
Concanavalin A makes a small contribution of kainate receptors to glutamate responses visible. ***A, B,*** Representative current traces recorded from GluA4-expressing (A) and GluA4-deficient horizontal cell somata (B) upon application of the glutamate and GYKI52466 while concanavalin A (ConA, 1 mg/ml), known to potentiate kainate receptor-mediated currents, was continuously present in Ringer’s solution. In both genotypes, glutamate induced a discernible current when AMPA receptors were blocked, suggesting a small contribution of kainate receptors to the overall glutamate response. ***C***, Quantification of glutamate-induced currents in the presence of ConA. In both genotypes, responses to glutamate in the presence of GYKI52466 and ConA were significantly higher than to Ringer’s application. Current amplitudes in C are normalized to 10 pF capacitance of cell membrane to account for differences in cell size. Small circles represent values for individual cells; large, black filled circles represent the means; error bars show SD. n specifies the number of measured cells. *, p < 0.05; **, p < 0.01.

Thus, in GluA4fl/fl controls, the AMPA receptor blocker GYKI52466 did not completely block glutamate-induced inward currents, pointing to a potential contribution of kainate or NMDA receptors. To test for kainate receptors, we exploited that kainate-induced currents can be potentiated by concanavalin A (ConA) [28]. Therefore, glutamate-induced currents were measured in the presence of GYKI52466 and 1 mg/ml ConA in the Ringer’s solution. Under these conditions, an inward current was discernible in both genotypes (Fig. 7A,B) which was significantly different from responses to Ringer’s application (Fig. 7C; GluA4fl/fl: p = 0.0168; GluA4fl/fl:Cx57+/Cre: p = 0.0047) and significantly larger than the GYKI52466-insensitive currents in normal Ringer’s (please compare Fig. 6 and 7; GluA4fl/fl: p = 0.0181; GluA4fl/fl:Cx57+/Cre: p = 6.9×10^−4^). These data indicate that dissociated horizontal cells in both genotypes contain kainate receptors which contribute very little to the overall glutamate response. However, this small contribution can be potentiated by bath application of ConA. When testing for a contribution of NMDA receptors, we did not detect any inward currents when NMDA (500 µM) was applied, even when glycine (1 µM) was co-applied and horizontal cells were clamped at –20 mV (not shown). This is consistent with previous reports on mouse and rabbit horizontal cells [9], [29].

In summary our electrophysiological data show that 1) glutamate-induced currents in horizontal cell somata of the mouse retina are largely mediated by AMPA receptors and only to a very small degree by kainate receptors, 2) deletion of the AMPA receptor subunit GluA4 decreases glutamate-induced currents by ∼75%, and 3) the residual 25% of the current is mostly mediated by AMPA receptors.

## Discussion

### Specificity of Cre expression in Cx57+/Cre mice

The mammalian retina contains more than 60 different neuronal cell types [30]. Several of these cell types have been targeted for Cre recombinase expression [31]–[34], e.g., the ChAT-cre/Jax knockin mouse line, expressing Cre under the choline acetyltransferase promoter, was found to exclusively target the cholinergic starburst amacrine cells in the retina [32] and proved a useful tool to study starburst arborization [35]. However, Cre recombinase targeting is not always specific, especially when bacterial artificial chromosomes (BAC) are used to introduce the Cre gene. The ChAT-cre/Gsat line, for example, generated using a BAC clone, does not show Cre expression in starburst cells but in non-cholinergic amacrine cells and in ganglion cells [32]. Here, we present a horizontal cell-specific Cre recombinase mouse line. Horizontal cells have been targeted for Cre expression before but Cre expression was not exclusive to horizontal cells because it occurred in retinal progenitor or precursor cells [36]–[39]. In our knockin line, the Cx57 promoter was used to drive Cre expression. Cx57 was reported to be only expressed in the embryonic kidney, the thymus medulla, and the retina [4] in which it is exclusively expressed in horizontal cells [6]. We replaced one allele of *Cx57* with Cre recombinase and as expected, Cre expression was highly specific; no other retinal cell type was found to express the enzyme in Cx57+/Cre mice. Also, Cre expression proved to be uniform and not patchy as often reported for Cre-expressing mouse lines [18], [32]: almost all (>99%) horizontal cells expressed Cre. The high specificity of the *Cx57* locus was already shown in the Cx57+/DTR mouse line (derived from the same targeting construct; Fig. 1A), which allows for selective ablation of horizontal cells from the adult retina [7]. Consistent with the presence of Cx57 in the adult thymus, Cre expression was also observed in medullary thymic epithelial cells [20].

### Glutamate receptor subunits in mouse horizontal cells

As, in the retina, Cre expression is restricted to horizontal cells, the Cx57+/Cre mouse line may be exploited to selectively ablate genes from these interneurons without affecting other retinal neurons. In this study, we used the GluA4fl/fl mouse [22] to test for Cre enzyme activity. In the rodent retina, GluA4 is expressed in horizontal cells, rod bipolar cells and OFF bipolar cells in the OPL [1] and some amacrine and many ganglion cells [1], [40], [41] in the IPL. Consistent with a selective deletion of GluA4 from Cre-expressing horizontal cells, Western blot analysis showed a reduction in the GluA4-immunoreactive protein in GluA4fl/fl:Cx57+/Cre mice. Also, immunohistochemistry revealed a significantly reduced (∼50%) GluA4 immunoreactivity in the OPL, where horizontal cell terminals receive glutamatergic inputs from photoreceptors. However, some GluA4-immunoreactive puncta remained in the OPL of GluA4fl/fl:Cx57+/Cre mice (Fig. 2I, J; Fig. S1E). Double labeling with the bipolar cell marker secretagogin which labels several types of ON and OFF cone bipolar cells in the mouse retina [42] showed that in both genotypes GluA4 immunoreactivity in the OPL is partially localized on OFF bipolar cell dendrites (Fig. S2G-L, arrowheads) as was suggested before [1]. We also examined the possibility that the remaining GluA4 signals in GluA4fl/fl:Cx57+/Cre mice originate from horizontal cells because the Cre recombinase has an efficiency of less than 100% [18]. High magnification images of the OPL of GluA4fl/fl:Cx57+/Cre mice revealed that the residual GluA4 immunoreactivity occasionally colocalized with calbindin-positive horizontal cell dendrites (Fig. S1A-F, arrowheads). Thus, Cre recombinase is expressed by >99% of all horizontal cells but may not be effective in all of them; such a less than 100% efficiency of the Cre recombinase was reported before [18].

In contrast to GluA4 immunoreactivity, label for the AMPA receptor subunits GluA2/3, known to be expressed in mouse horizontal cells [1], was unaffected in the OPL of GluA4-deficient mice, consistent with the high specificity of Cre recombinase expression. As expected for a mouse line lacking one Cx57 allele, Cx57 immunoreactivity was significantly reduced in GluA4fl/fl:Cx57+/Cre mice. It is important to note that decreased Cx57 alone might induce changes in this mouse line because the lack of gap junctions might influence the development of the outer retina or horizontal cell physiology. However, electron microscopy revealed that the synaptic triads between photoreceptors, ON bipolar and horizontal cells are normal in Cre-expressing mice (Fig. S2A-D). This is in line with earlier reports that retina development is not impaired and the horizontal cell mosaic is normal in Cx57-deficient mice [4], [5]. Shelley et al. (2006) reported an increase in the resting potential, a decrease in input resistance, suggesting the upregulation of an ion channel, and a decrease in coupling in Cx57-deficient horizontal cells. However, we did not find any differences in the resting potential of dissociated horizontal cells from control and Cre-expressing mice (GluA4fl/fl: –30 ±17 mV; GluA4fl/fl:Cx57+/Cre: –34 ±18 mV; n = 11 for GluA4fl/fl and n = 9 for GluA4fl/fl:Cx57+/Cre; p = 0.6201) suggesting that the properties of horizontal cells in Cx57+/Cre mice are not altered.

In summary, the strong reduction in GluA4 immunoreactivity verified that the Cre enzyme is active and able to excise the floxed GluA4 coding sequence from horizontal cells. Thus, we used this mouse line to study the contribution of the GluA4 subunit to glutamate-induced inward currents in horizontal cells.

AMPA receptors are tetramers formed by the combination of one or more of the four closely related subunits GluA1-4 (formerly GluR1-4) [43]. They allow the passage of sodium, potassium and, in the absence of the GluA2 subunit, calcium ions [44]. Ablation of GluA4 from horizontal cells led to a reduction in glutamate-induced inward currents of about 75%. This is in line with earlier physiological studies from the rabbit [29], [45] and the mouse retina [9] which indicated that horizontal cell responses to glutamate receptor agonists are mainly mediated by AMPA receptors. The residual glutamate response in GluA4-deficient horizontal cells showed a similar glutamate dependency as responses from wild-type cells, indicating that the remaining glutamate receptors have similar properties as wild-type receptors. Blockade with the AMPA receptor antagonist GYKI52466 completely abolished glutamate- or AMPA-induced currents in GluA4-deficient mice while a very small current was still detectable in controls. Incubation with ConA, a substance known to potentiate kainate receptor-mediated currents [28], revealed a small contribution of kainate receptors in both genotypes: when ConA was present GYKI-insensitive currents were significantly larger than in normal Ringer’s. Such a small contribution of kainate receptors is consistent with previous reports showing the expression of the kainate receptor subunits GluK2/3 (formerly GluR6/7) on rodent horizontal cells [1], [24] and with physiological studies on isolated mammalian horizontal cells indicating the presence of kainate receptors [9], [45].

In both genotypes, we were not able to induce currents by the application of NMDA. Depolarizing horizontal cells and simultaneous application of NMDA and the allosteric activator glycine did not induce any detectable currents. Likewise, other groups failed to detect any NMDA-induced calcium signals in isolated mouse [9] or rabbit horizontal cells [29]. As metabotropic glutamate receptors were never found on mammalian horizontal cells [46]–[49], a contribution of these receptors to the horizontal cell glutamate response seems also unlikely.

Shelley et al. (2006) reported a decrease in input resistance in dissociated horizontal cells from Cx57lacZ/lacZ mice when compared to wild-type cells. The same authors also demonstrated that horizontal cells heterozygous for Cx57 show light response amplitudes and dark resting potentials midway between wild-type and homozygous knockout cells [5]. Their results suggest the upregulation of a conductance as a compensation for the lack of Cx57, adding a layer of difficulty in interpreting effects of Cre-mediated excision of GluA4. However, apart from the 75% reduction in glutamate-induced currents, horizontal cells from mice with one or two alleles of Cx57 behaved very similar showing the same glutamate dependence and the same mean resting membrane potential (see above). Hence, our data suggest that lack of Cx57 does not confound the physiology of dissociated horizontal cells.

Thus, with the help of the GluA4fl/fl:Cx57+/Cre mouse line, we were able to show for the first time that the AMPA receptor subunit GluA4 makes the major contribution to glutamate responses of dissociated mouse horizontal cells. It will be interesting to see how GluA4-deficient horizontal cells, which receive less excitatory input, influence the receptive fields of bipolar or ganglion cells.

### Cx57-Cre as a genetic tool for studying horizontal cells

The Cre/*loxP* recombination system has become a useful tool for cell type-specific gene deletion, ectopic gene expression and over-expression [32]. A cell type-specific promoter (here Cx57) is used to drive Cre expression, allowing the ablation of a gene whose systematic absence is lethal [19] or which is rather broadly expressed in the tissue of interest (here GluA4). The efficiency of the Cre/*loxP* system mainly depends on the promoter. The Cx57 promoter we used here is strong enough to drive Cre expression in almost all horizontal cells allowing the deletion of genes from almost the entire horizontal cell network. As the promoter is active from embryonic day 16.5 on [4], genes can be specifically ablated from horizontal cells before birth. Thus, the Cx57+/Cre line makes it possible to selectively eliminate ion channels and transporters, neurotransmitter receptors, e.g. the dopamine receptor D1 subunit [50], or proteins of the putative GABA release machinery, e.g. the vesicular GABA transporter [51] or SNAP-25 [52], from horizontal cells. Despite its lack of one allele of *Cx57*, it provides a versatile tool, for example to reveal the nature of horizontal cell feedback and feedforward signals or to dissect the mechanisms of horizontal cell transmitter release [2].

## Materials and Methods

Unless stated otherwise, all chemicals were purchased from Carl Roth GmbH (Karlsruhe, Germany).

### Ethics statement

All experiments were carried out in accordance with the institutional guidelines for animal welfare of the University of Oldenburg, following the standards described by the German animal protection law (*Tierschutzgesetz*). The mere killing of mice for tissue analysis is registered with the local authorities (*Niedersächsisches Landesamt für Verbraucherschutz und Lebensmittelsicherheit*) and reported on a regular basis as demanded by law but needs no further approval if no other treatment is applied before killing.

### Generation and genotyping of mice

The Cx57-Cre recombinase strain (Cx57+/Cre) was generated as described (Fig. 1) [7]. Briefly, a part of Cx57 exon 2 was exchanged by an *frt*-flanked DTR-eGFP cassette, which was followed by the coding sequence of nuclear localization sequence (NLS)-Cre recombinase. The *frt*-flanked DTR-eGFP cassette was then excised *in vivo* by mating to Flp-deleter mice (*hACTB:FLPe* mice, [53]). This brought the Cre recombinase under direct control of the *Cx57* promoter and left a single *frt* site in intron 1. Cx57-DTRfrtCre mice can be obtained from the European Mouse Mutant Archive (http://www.emmanet.org/ mutant_types.php?keyword = EM:06024). Mice lacking GluA4 expression in Cx57-expressing horizontal cells were produced by breeding mice carrying a *loxP*-flanked exon 11 of the GluA4 allele (GluA4fl/fl; generously provided by Dr. Elke Fuchs and Prof. Hannah Monyer, University of Heidelberg, Heidelberg, Germany) [22] with the Cx57+/Cre mouse line. Both lines were generated in a C57Bl/6 genetic background.

Mice were genotyped for genes encoding Cx57, GluA4, and Cre recombinase by polymerase chain reaction (PCR) analysis of tail DNA using sets of primers listed in Table 1. To distinguish the different *Cx57* alleles, a three primer PCR was used in which a *Cx57*-specific forward primer was combined with reverse primers for Cx57 or Cre recombinase (Table 1). PCR conditions were as follows: 5 min at 94°C, 94°C for 45 s, 63°C for 1 min, 72°C for 1 min (25 cycles), 72°C for 7 min, resulting in a 720 bp amplicon for the Cx57 wild-type allele and a 980 bp amplicon for the Cx57-Cre allele.

**Table 1:** Primers used for mouse genotyping.

Primer	Primer sequence 5‵ to 3′	References*
Cx57 for	CAA TGA GTG GTA GTG GAA GCT TAG	[7]
Cx57 rev	GGC CCA TAT ACA CCA AAG AAG GG	[7]
intCre rev	TCC ATG AGT GAA CGA ACC TGG TCG	
ScaI for	GGT AAG AGC AGC AAA TAC CC	
ScaI rev	CTG GTT GCT CTT CTC AGT TC	
EF511	CAC TAT GTC TCA GTT CTC TCA AG	[22]
EF188	ACG ATT GCA ACT AAG TTC ACA C	[22]

related to primer sequences and/or PCR conditions used.

Mice harboring the *loxP*-flanked GluA4 were genotyped using the primer combination EF511 and EF188 (see Table 1). PCR conditions were as follows: 5 min at 94°C, 94°C for 30 s, 53°C for 30 s, 72°C 30 s (35 cycles), 72°C for 5 min, resulting in a 380 bp amplicon for the GluA4 wild-type allele and a 450 bp amplicon for the floxed GluA4 allele.

### Southern blot analysis

For southern blot hybridization genomic DNA from mouse liver was digested using the restriction enzyme ScaI and blotted onto a Hybond-N^+^ membrane (GE Healthcare, Freiburg, Germany). Hybridization was performed using QuickHyp® (Stratagene, Heidelberg, Germany) according to the instruction of the manufacturer. A 508 bp probe corresponding to a region in intron 2 of Cx57 was generated using the primers ScaI for and ScaI rev (see primer list). A fragment of 6.6 kb is generated for the wild-type allele, whereas the targeted alleles are indicated by respective signals of 7.2 kb (Cx57-DTRfrtCre) and 8.7 kb (Cx57-Cre).

### Animals and tissue preparation

Mice were kept under a 12 h light/dark cycle with food and water *ad libitum* and were used for experiments at ages between 1.5 and 6 months (6–10 weeks for patch-clamp recordings). Mice were anesthetized with CO_2_ and killed by cervical dislocation. Each eye was enucleated and transferred to a dish with carboxygenated (95% O_2_ / 5% CO_2_) extracellular solution containing (mM) 110 NaCl, 2.5 KCl, 1 CaCl_2_, 1.6 MgCl_2_, 10 D-glucose, 22 NaHCO_3_, pH 7.4). The eye was opened and cornea, lens and vitreous were removed, leaving the retina in the posterior eyecup.

### Western blot analysis

SDS-PAGE and Western blot analysis were carried out as described [54]. Briefly, retinas were homogenized in buffer containing (mM) 50 Tris-HCl (pH 7.4), 2 ethylene glycol-bis(2-aminoethylether)-N,N,N’,N’-tetraacetic acid, 2 ethylenediaminetetraacetic acid, 0.1 sodium orthovanadate, 1 dithioerythritol, 1 phenylmethylsulfonyl-fluoride, protease (complete Mini EDTA-free, Roche) and phosphatase inhibitor cocktail (PhosphoSTOP, Roche). Samples of total homogenates (50 µg of protein) were separated according to their molecular weight by SDS-PAGE on 8–10% gradient gels. Proteins were transferred overnight (40 V at 4°C) to a nitrocellulose membrane (PROTRAN, Whatman, Dassel, Germany). Non-specific binding sites were blocked for 1 hour with 5% powdered milk in TBS-Tween (pH 7.4; 20 mM Tris-HCl, 150 mM NaCl, 0.2% Tween-20) at 37°C. The blot was then incubated with antibodies against GluA4 (polyclonal, rabbit, 1:000; Millipore, Bedford, MA). After several washing steps, secondary goat anti-rabbit IgG, conjugated to horseradish peroxidase (1:3,000 in 2% powdered milk in TBS-Tween; BioRad Laboratories, Munich, Germany), were applied for 2 hours. After washing, immunoreactive proteins were visualized using a chemiluminescence detection system (Pierce ECL; Thermo Fisher Scientific, Rockford, IL) following the manufacturer’s instructions. Bound antibodies were removed by incubation in two stripping buffers: 1) 10 mM Tris/HCl, pH 8.8, 1% SDS, 10 mM β-mercaptoethanol, and 2) 100 mM sodium citrate, pH 3.0, 1% SDS, 10 mM β-mercaptoethanol. After rinsing in TBS-Tween, blots were blocked again and re-probed with antibodies against α-tubulin (monoclonal, mouse, 1:5,000; Sigma, Deisenhofen, Germany).

For densitometric quantification of GluA4 and α-tubulin immunoreactivity, blots were scanned using an AlphaImager HP and the associated AlphaView program with its tool Spot Densor (Biozym Scientific, Hessisch-Oldendorf, Germany). For each sample, integrated density values (IDV) of the GluA4- and α-tubulin-immunoreactive protein bands were quantified in an area of 5,600 µm^2^ encompassing the band. Background was subtracted, and then the IDV measured in the GluA4 band was normalized to the IDV measured in the reference α-tubulin band. Values are given as mean ± standard deviation (SD) and were tested for statistical differences using the unpaired t-test in Excel (Microsoft Corporation, Redmond, WA, USA).

### Immunohistochemistry

Immunohistochemical labeling was carried out using the indirect fluorescence method as described previously [55]. For vertical cryosections, eyecups were fixed in 2% paraformaldehyde (PFA; Riedel de Haën, Seelze, Germany) in 0.1 M phosphate buffer (PB, pH 7.4) for 20 min. After washes in PB, eyecups were cryoprotected in 30% sucrose in PB overnight at 4°C and embedded in Cryoblock (Medite GmbH, Burgdorf, Germany). Vertical sections (18–25 µm) were cut on a cryostat (Bright, Huntingdon, UK) and were blocked with 5% donkey serum or 3% normal goat serum + 1% bovine serum albumin (BSA) in PB containing 0.3% Triton X-100. Primary antibodies (see below) were applied in blocking solution overnight at 4°C. After several washes in PB, slides were incubated with secondary antibodies for two hours at room temperature. Secondary antibodies were conjugated to Alexa Fluor 488 or 568 (1∶500; Invitrogen, Karlsruhe, Germany). After washing, sections were mounted in Vectashield (Vector Laboratories, Burlingame, CA).

For retinal whole-mounts, retinas were dissected from the eyecup, mounted on black filter paper (Millipore) and fixed in 4% PFA in PB for 20 min. After several washing steps, whole-mounts were blocked overnight with 3% normal goat serum + 1% BSA + 0.02% NaN_3_ in PB containing 0.75% Triton X-100. Primary antibodies were applied in the same solution for three days. After rinsing, secondary antibodies conjugated to FITC, Alexa Fluor 488 or 568 were applied overnight. Whole-mounts were washed and finally mounted in Vectashield.

For controls, the primary antibody was omitted; strong labeling of blood vessels was observed for secondary anti-mouse antibodies.

### Primary antibodies for immunohistochemistry

The following primary antibodies were used in immunohistochemistry: polyclonal rabbit anti-Cre recombinase (1∶3,000; Covance Research Products, Berkeley, CA), monoclonal mouse anti-calbindin (1∶2,000; Swant, Bellinzona, Switzerland), polyclonal rabbit anti-Cx57 (1:500) [6], polyclonal rabbit anti-GluA2/3 (1∶100; Millipore), polyclonal rabbit anti-GluA4 (1:100; Millipore), and polyclonal sheep anti-secretagogin (1∶2,000; BioVendor).

### Electron microscopy

For electron microscopy, retinas from both genotypes were fixed in 2% paraformaldehyde, 2.6% glutaraldehyde, and 3% sucrose in 0.1 M PB overnight at 4°C. The tissue was post-fixed in 1% OsO_4_ in PB. After dehydration in a series of 30% to 100% acetone, retinas were embedded in AGAR 100 resin (AGAR Scientific, Essex, UK). 90 nm ultrathin sections were cut with a Reichert-Jung Ultracut E and analyzed using a Zeiss 902 electron microscope.

### Image acquisition and quantification

Retinas from the same experimental group were prepared, incubated, and scanned with a Leica TCS SL (SP2 or SP5; Leica Microsystems, Wetzlar, Germany) under identical conditions. Images were taken with 40x (NA 1.25 or 1.3) or 63x (NA 1.32) immersion objectives. If not stated otherwise, images are presented as maximum projections of collapsed confocal stacks. Images were adjusted in brightness and contrast for presentation purposes using ImageJ (http://rsbweb.nih.gov/ij/).

To determine the number of Cre recombinase- and calbindin-positive cells in retinal whole-mounts, the *Point picker* plugin in ImageJ was used. We sampled 11 fields (258×258 µm^2^) from three whole-mount retinas (from two Cx57+/Cre mice) and 16 fields from four whole-mount retinas (from two GluA4fl/fl:Cx57+/Cre mice), approximately 400 µm distant from the optic nerve head, and counted the immunolabeled somata in maximum projections of confocal stacks.

To quantify the density of Cx57-, GluA2/3-, and GluA4-positive puncta in the outer plexiform layer (OPL) of GluA4fl/fl and GluA4fl/fl:Cx57+/Cre mice, we used the A*nalyze particle* plugin in ImageJ as we described earlier [6], [56]. Cryosections were double-labeled with calbindin and either Cx57, GluA2/3 or GluA4. In maximum projections of two confocal sections (thickness 0.4 µm), regions of interest (ROI; 79×19 µm^2^) were defined. To ensure an accurate count, an intensity threshold was set manually and a size threshold was fixed (2×2 pixels) to avoid inclusion of noise. Thresholds were the same for all ROIs from the same experimental group. The number of plaques and the fraction of the area these plaques filled in the respective ROI were averaged from 6-10 ROIs, obtained from 2–3 mice for each genotype.

All values are given as mean ± SD and were compared for statistical differences using the unpaired t-test in Matlab R2009a (MathWorks, Ismaning, Germany).

### Horizontal cell dissociation

The dissociation of horizontal cells was performed as described previously [10]. Briefly, retinas were dissected from the eyecups and stored in Hank’s Balanced Salt Solution (HBSS; Biochrom, Berlin, Germany). Then both retinas were dissociated enzymatically with the help of a papain solution (20 U/ml in HBSS; Worthington Biochemical, Freehold, NJ). The reaction was stopped after 19 to 25 minutes with Dulbecco’s Modified Essential Medium (DMEM, Biochrom) containing DNAse I (100 U/ml; Sigma, Deisenhofen, Germany) and fetal calf serum (Biochrom). Predigested retinas were transferred into DMEM and triturated four times using fire-polished Pasteur pipettes with decreasing open diameter. Three fractions with horizontal cells were pooled and then seeded on glass coverslips coated with ConA (1 mg/ml; Sigma). The cells were then stored in an incubator at 55% O_2_ / 5% CO_2_ and 36°C and allowed to settle for at least 30 min prior to recording.

### Patch-clamp recordings from isolated horizontal cells

For recordings, the Ringer’s solution contained (mM) 137 NaCl, 5.4 KCl, 1.8 CaCl_2_, 1 MgCl_2_, 10 D-glucose, and 5 HEPES (pH 7.4). Drug solutions were freshly prepared on each day from stock solutions in TC Water (PromoCell, Heidelberg, Germany). α-amino-3-hydroxy-5-methyl-4-isoxazolepropionic acid (AMPA) and 1-(4-aminophenyl)-4-methyl-7,8-methylenedioxy-5H-2,3-benzodiazepine GYKI52466 (GYKI) were purchased from Tocris (Bristol, UK), glutamate and N-methyl-D-aspartate (NMDA) from Sigma, and glycine from Fluka (Deisenhofen, Germany).

Glass coverslips with horizontal cells were transferred into the recording chamber (Luigs and Neumann, Ratingen, Germany) on an upright microscope (Leica) and cells were kept in Ringer’s solution. Horizontal cells were visually identified according to their unique morphology using a 40x water immersion objective. Patch pipettes were pulled from borosilicate glass (Hilgenberg, Malsfeld, Germany) with a horizontal electrode puller (Sutter, Novato, CA); the resistance of these electrodes ranged from 3 to 6 MΩ. Electrodes were filled with intracellular solution containing (mM) 120 CsCl, 1 CaCl_2_, 2 MgCl_2_, 20 TEA-Cl, 11 EGTA, and 10 HEPES (pH 7.2). Currents were recorded in the whole-cell configuration and voltage-clamp mode of an EPC9 double patch-clamp amplifier (Heka, Lambrecht, Germany) at a sampling rate of 1–20 kHz. Unless stated otherwise, drugs were applied by an air pressure-driven application system (DAD-12 superfusion system, ALA Scientific Products, New York, NY) mounted to a second micromanipulator. When applying glutamate receptor antagonists, the time between two successive applications was set to the minimal value of 20 ms.

### Data analysis

For data analysis, the recorded steady state current before drug application was compared to the corresponding steady state current during drug application, using custom-written Matlab scripts (R2011b). Dose response curves were created using the following equation:



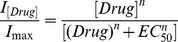
,

where 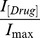
is the normalized current for a given drug concentration, 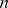 is the Hill coefficient and 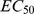 is the drug concentration for the half maximal current amplitude. Values are given as mean ± SD if not stated otherwise and were compared for statistical differences using the unpaired t-test in Matlab.

## Supporting Information

Figure S1 **OFF bipolar and few horizontal cell dendrites show GluA4 immunoreactivity in GluA4fl/fl:Cx57+/Cre mice.** ***A-F,*** Double labeling for calbindin and GluA4 in GluA4fl/fl (A-C) and GluA4fl/fl:Cx57+/Cre mice (D-F). GluA4 immunoreactivity shows large and bright patches in the OPL of GluA4fl/fl mice (B). Most patches are associated with calbindin-positive horizontal cell dendrites (C, inset, arrowhead) and only a few patches are not (C, inset, arrow). In contrast, GluA4-immunoreactive puncta appear smaller and are less bright and numerous in GluA4fl/fl:Cx57+/Cre mice (E) in which they occasionally colocalize with calbindin-positive horizontal cell dendrites (F, inset, arrowheads). However, most remaining GluA4-positive puncta are not colocalized with calbindin (F, inset, arrow). ***G-I,*** Double labeling for GluA4 and secretagogin, a bipolar cell marker, in GluA4fl/fl (G-I) and GluA4fl/fl:Cx57+/Cre mice (J-L). GluA4-immunoreactive puncta colocalize with secretagogin-positive OFF bipolar cell dendrites in the proximal OPL (I, L, insets, arrowheads) in both genotypes. All images are maximum projections of three confocal images (0.51 µm). Dashed squares are shown enlarged as insets. Scale bars: D, J: 10 µm; F, L: 2.5 µm. INL, inner nuclear layer; ONL, outer nuclear layer; OPL, outer plexiform layer.
(PDF)

Figure S2 **Synaptic triads of rods and cones are intact in GluA4fl/fl:Cx57+/Cre.** Electron micrographs of the outer plexiform layer of GluA4fl/fl (A, C) and GluA4fl/fl:Cx57+/Cre mice. Synaptic triads of rods (A, B) and cones (C, D) show no differences and contain lateral elements (asterisks), formed by horizontal cell dendrites, in both genotypes. Scale bar: 1 µm.
(PDF)
