# Supplementary material for: Cell-Specific Cre Recombinase Expression Allows Selective Ablation of Glutamate Receptors from Mouse Horizontal Cells
Source: PLoS One. 2013 Dec 12;8(12):e83076. doi: 10.1371/journal.pone.0083076 (PMC3861464; doi:10.1371/journal.pone.0083076)
Supplement: Figure S1 — OFF bipolar and few horizontal cell dendrites show GluA4 immunoreactivity in GluA4fl/fl:Cx57+/Cre mice. A-F, Double labeling for calbindin and GluA4 in GluA4fl/fl (A-C) and GluA4fl/fl:Cx57+/Cre mice (D-F). GluA4 immunoreactivity shows large and bright patches in the OPL of GluA4fl/fl mice (B). Most patches are associated with calbindin-positive horizontal cell dendrites (C, inset, arrowhead) and only a few patches are not (C, inset, arrow). In contrast, GluA4-immunoreactive puncta appear smaller and are less bright and numerous in GluA4fl/fl:Cx57+/Cre mice (E) in which they occasionally colocalize with calbindin-positive horizontal cell dendrites (F, inset, arrowheads). However, most remaining GluA4-positive puncta are not colocalized with calbindin (F, inset, arrow). G-I, Double labeling for GluA4 and secretagogin, a bipolar cell marker, in GluA4fl/fl (G-I) and GluA4fl/fl:Cx57+/Cre mice (J-L). GluA4-immunoreactive puncta colocalize with secretagogin-positive OFF bipolar cell dendrites in the proximal OPL (I, L, insets, arrowheads) in both genotypes. All images are maximum projections of three confocal images (0.51 µm). Dashed squares are shown enlarged as insets. Scale bars: D, J: 10 µm; F, L: 2.5 µm. INL, inner nuclear layer; ONL, outer nuclear layer; OPL, outer plexiform layer. (PDF) [file pone.0083076.s001.pdf]

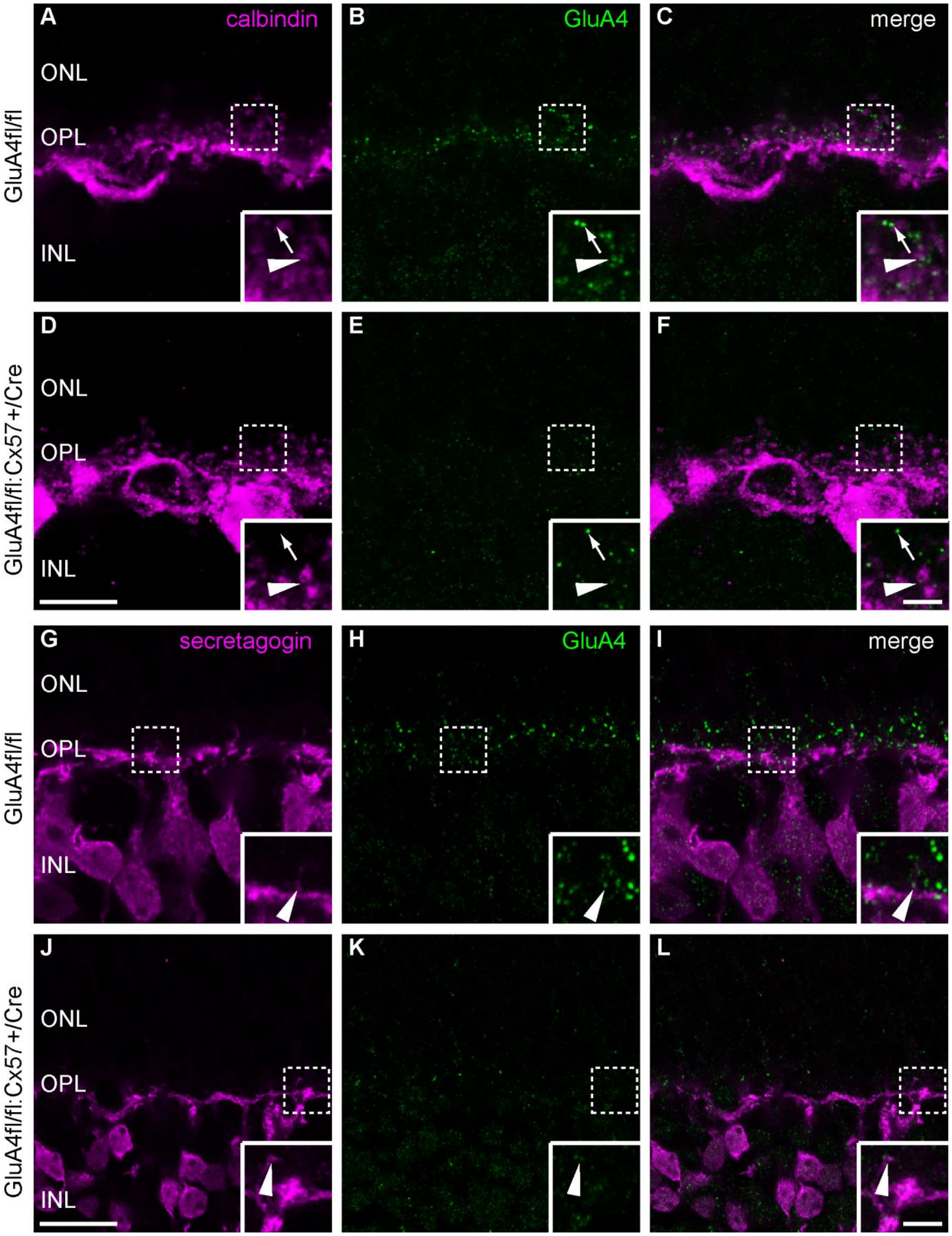

Figure S1. OFF bipolar and few horizontal cell dendrites show GluA4 immunoreactivity in GluA4fl/fl:Cx57+/Cre mice.

A-F, Double labeling for calbindin and GluA4 in GluA4fl/fl (A-C) and GluA4fl/fl:Cx57+/Cre mice (D-F). GluA4 immunoreactivity shows large and bright patches in the outer plexiform

layer of GluA4fl/fl mice (B). Most patches are associated with calbindin-positive horizontal cell dendrites (C, inset, arrowhead) and only a few patches are not (C, inset, arrow). In contrast, GluA4-immunoreactive puncta appear smaller and are less bright and numerous in GluA4fl/fl:Cx57+/Cre mice (E) in which they occasionally colocalize with calbindin-positive horizontal cell dendrites (F, inset, arrowheads). However, most remaining GluA4-positive puncta are not colocalized with calbindin (F, inset, arrow).

G-I, Double labeling for GluA4 and secretagogin, a bipolar cell marker, in GluA4fl/fl (G-I) and GluA4fl/fl:Cx57+/Cre mice (J-L). GluA4-immunoreactive puncta colocalize with secretagogin-positive OFF bipolar cell dendrites in the proximal OPL (I, L, insets, arrowheads) in both genotypes. All images are maximum projections of three confocal images (0.51 µm). Dashed squares are shown enlarged as insets. Scale bars: D, J: 10 µm; F, L: 2.5 µm.

INL, inner nuclear layer; ONL, outer nuclear layer; OPL, outer plexiform layer.
