# Supplementary material for: Cell-Specific Cre Recombinase Expression Allows Selective Ablation of Glutamate Receptors from Mouse Horizontal Cells
Source: PLoS One. 2013 Dec 12;8(12):e83076. doi: 10.1371/journal.pone.0083076 (PMC3861464; doi:10.1371/journal.pone.0083076)
Supplement: Figure S2 — Synaptic triads of rods and cones are intact in GluA4fl/fl:Cx57+/Cre. Electron micrographs of the outer plexiform layer of GluA4fl/fl (A, C) and GluA4fl/fl:Cx57+/Cre mice. Synaptic triads of rods (A, B) and cones (C, D) show no differences and contain lateral elements (asterisks), formed by horizontal cell dendrites, in both genotypes. Scale bar: 1 µm. (PDF) [file pone.0083076.s002.pdf]

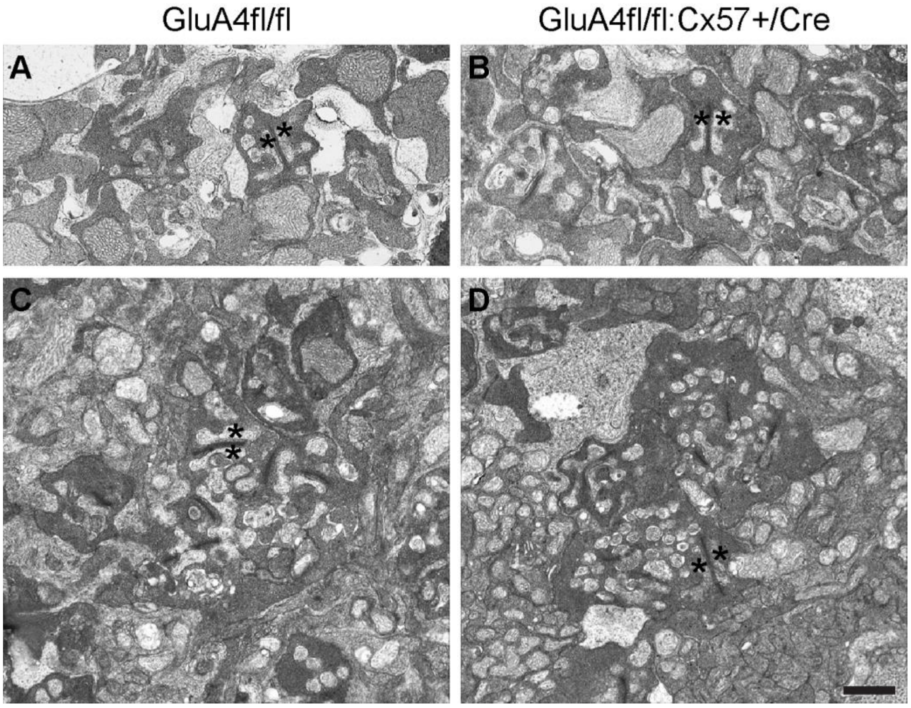

Figure S2. Synaptic triads of rods and cones are intact in GluA4fl/fl:Cx57+/Cre. Electron micrographs of the outer plexiform layer of GluA4fl/fl (A, C) and GluA4fl/fl:Cx57+/Cre mice. Synaptic triads of rods (A, B) and cones (C, D) show no differences and contain lateral elements (asterisks), formed by horizontal cell dendrites, in both genotypes. Scale bar: 1 µm.
